# Longitudinal saliva omics responses to immune perturbation: a case study

**DOI:** 10.1038/s41598-020-80605-6

**Published:** 2021-01-12

**Authors:** George I. Mias, Vikas Vikram Singh, Lavida R. K. Rogers, Shuyue Xue, Minzhang Zheng, Sergii Domanskyi, Masamitsu Kanada, Carlo Piermarocchi, Jin He

**Affiliations:** 1 Biochemistry and Molecular Biology, Michigan State University, East Lansing, MI 48824 USA; 2 Institute for Quantitative Health Science and Engineering, Michigan State University, East Lansing, MI 48824 USA; 3 Microbiology and Molecular Genetics, Michigan State University, East Lansing, MI 48824 USA; 4 Physics and Astronomy, Michigan State University, East Lansing, MI 48824 USA; 5 Pharmacology and Toxicology, Michigan State University, East Lansing, MI 48824 USA

**Keywords:** Molecular medicine, Diagnostic markers, Transcriptomics, Gene expression, Data integration

## Abstract

Saliva omics has immense potential for non-invasive diagnostics, including monitoring very young or elderly populations, or individuals in remote locations. In this study, multiple saliva omics from an individual were monitored over three periods (100 timepoints) involving: (1) hourly sampling over 24 h without intervention, (2) hourly sampling over 24 h including immune system activation using the standard 23-valent pneumococcal polysaccharide vaccine, (3) daily sampling for 33 days profiling the post-vaccination response. At each timepoint total saliva transcriptome and proteome, and small RNA from salivary extracellular vesicles were profiled, including mRNA, miRNA, piRNA and bacterial RNA. The two 24-h periods were used in a paired analysis to remove daily variation and reveal vaccination responses. Over 18,000 omics longitudinal series had statistically significant temporal trends compared to a healthy baseline. Various immune response and regulation pathways were activated following vaccination, including interferon and cytokine signaling, and MHC antigen presentation. Immune response timeframes were concordant with innate and adaptive immunity development, and coincided with vaccination and reported fever. Overall, mRNA results appeared more specific and sensitive (timewise) to vaccination compared to other omics. The results suggest saliva omics can be consistently assessed for non-invasive personalized monitoring and immune response diagnostics.

## Introduction

Precision medicine continues its rapid development toward clinical applications aided by new sequencing technology and computational capabilities. Major efforts have concentrated on evaluating disease risk from genomic information^1,2^, including direct to consumer platforms, like 23andMe^3^, as well as pharmacogenomic evaluations^4^. Implementing omics profiling in the clinic will require evaluation of patients over time. The utility of such profiling has been evaluated in individual monitoring pioneered in the integrative Personal Omics Profiling (iPOP) study^5^, and expanded recently to include profiling using electronic health devices^6^. The Pioneer study^7^ also incorporated behavioral coaching to improve clinical biomarkers based on participants’ individual data. Additional developments have included utilizing host–microbiome data in insulin resistant individuals in a study of weight gain^8^ and in prediabetics^9^, investigating biological age^10,11^ as well as monitoring of astronauts in the recent NASA twin study^12^.

In this investigation we are extending integrative omics to evaluate the utility of such monitoring using saliva. There has been long-standing interest in saliva for non-invasive diagnostics and health monitoring, and saliva omics is an emerging field, with broad profiling that includes total saliva RNA and proteomes, as well as cell-free RNA identification, extracellular vesicle (EV) profiling, miRNAs as biomarkers, and salivary microbiomes^13–25^. Utilizing saliva for non-invasive monitoring is important in evaluating vulnerable populations, including infants, children, older adults and immunocompromised individuals. Additionally, saliva is important in evaluation of health in remote or underserved locations, when limited resources are available, where processing of blood samples might not be feasible, or a physician may not even be available. Such monitoring is also of particular interest for evaluating active personnel, including astronauts in deep space missions. The recent twin astronaut study evaluated multi-omics utility but also highlighted the logistic issues of using blood samples when these cannot be processed on-site^12^. The COVID-19 pandemic has additionally ignited interest in the use of saliva for rapid diagnostics, towards a rapid and minimally invasive diagnostic that can be used without risk to personnel (possibly as a home-use kit), including profiling viral loads (from posterior oropharyngeal samples)^26^, and current work continues to evaluate the sensitivity of saliva for practical implementations^27–31^.

**Figure 1:**
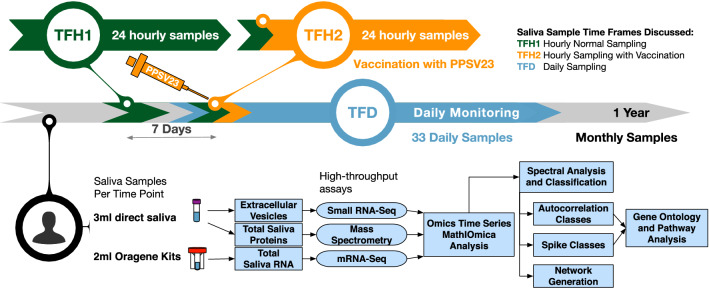
Study overview. The study followed an individual over the span of a year, collecting 100+ saliva samples. In this manuscript we discuss three time frames of interest for saliva sampling: (1) TFH1, where 24 hourly samples were taken, (2) TFH2, where 24 hourly samples were taken but the subject was also vaccinated with PPSV23 during this time frame, (3) TFD, where daily samples were taken. At each timepoint unstimulated saliva was collected (at a rate $\sim 500 \,\upmu \,\hbox {l/min}$). 3 ml of saliva were stored directly and subsequently used to extract extracellular vesicle RNA, and proteins for mass spectrometry proteome profiling. Furthermore, 2 ml saliva was collected with Oragene (DNAGenotek) kits, which contain a stabilizer, and used to profile total saliva transcriptomics using RNA-sequencing; see “Methods” for further collection and processing details. The data were used to generated time series with MathIOmica, which revealed multiple trends corresponding to response to immunization with PPSV23.

We are carrying out a clinical trial monitoring individualized response to pneumococcal vaccination, and in a proof-of-principle case-study, we monitored individualized response to the standard 23-valent pneumococcal polysaccharide vaccination (PPSV23), in a generally healthy individual (Caucasian male, 38, has reported chronic sinusitis), and carried out integrative profiling on saliva pre- and post-vaccination with pneumococcal PPSV23 vaccine. This is to our knowledge the most extensive saliva-focused omics dataset on an individual, covering 104 timepoints over one year. The period covers a healthy period as well as monitoring of innate and adaptive immune responses following vaccination. Protein and RNA from saliva were produced over 100 timepoints over the course of 1 year, and comprehensive untargeted proteomics and RNA-sequencing were carried out for all samples. The saliva sampled timepoints included three periods of particular reference in this manuscript: (1) 24 h hourly sampling without intervention to assess a healthy hourly baseline, (2) 24 h hourly sampling that included vaccination with pneumococcal vaccine (PPSV23) to assess response to the vaccine, (3) daily sampling following the vaccination to assess potential innate and adaptive immune responses reflected in the molecular saliva components.

Our study reveals multiple changes in response to pneumococcal vaccination that are observable in saliva. The microscopic collective behavior of multiple omics reflects physiological changes associated with immune response, including fever, innate and adaptive responses profiled over multiple scales. This case study provides a resource for future saliva studies, towards more effective non-invasive diagnostics.

## Results

### Samples and assays

We followed a single individual (m, 38, Caucasian), in general good health (has reported chronic sinusitis) over the span of a year. To observe whether the effects of perturbation can be profiled in saliva we carried out the profiling over 3 time frames. In the first 24 h time frame (TFH1) we established a baseline, obtaining a saliva sample from the subject hourly without perturbation over his standard routine. In the second 24 h time frame (TFH2), the subject was vaccinated with pneumococcal polysaccharide vaccine (PPSV23) within 3.5 h of waking up (at 10.30 am), while otherwise maintaining a similar routine as in the first period (including food intake and meal timing), and again saliva samples were taken hourly. We should note here that the subject reported fever $\sim$ 7.5 h post the vaccination (between timepoints at 5 and 6 pm), lasting for about 4 h (10 pm). The two time periods, TFH1 and TFH2, were treated as paired and combined in the analysis below ($\hbox {TF}\Delta$) to identify changes induced by the vaccination, by effectively removing daily normal routine effects for this individual. Additionally, in the third time frame (TFD) we monitored the subject daily for over a month, pre- and post-vaccination to identify potential immune changes over both innate and adaptive time frames (Fig. 1).

The daily samples were all taken at 8 am, to limit variability. Unstimulated saliva was collected both for downstream total RNA profiling, mass spectrometry proteomics, as well as for extraction of extracellular vesicles which were profiled for various small RNA molecular species (both host and non-host)—see “Methods” for further sample details.

### Analysis of total saliva and time series constructions

#### Total saliva transcriptomics

We profiled the transcriptome from total saliva at all timepoints using RNA-sequencing (RNA-seq) on extracted mRNA (150 bp paired-end reads, stranded). We mapped the RNA-seq data using Kallisto^32,33^, and adjusted the values across timepoints using sleuth^34^(DESeq^35^ adjustment of Transcripts per Million[aTPM]), resulting in 67,319 GENCODE^36^ annotation transcripts showing non-zero values for at least 3 timepoints (81,098 observed in at least 1 timepoint). We carried out downstream analysis in MathIOmica^37^, and selected the different timepoints for each of the three time frames. We tagged 0 aTPM values as Missing, filtered for noise (aTPM < 1), removed transcripts with more than 1/4 timepoints missing (i.e. reported as zero), and transcripts with constant values across all timepoints, to finally obtain 15,621, 7493, and 8155 transcript time series of aTPM values for TFH1, TFH2, and TFD respectively. All values were normalized to a reference—using the first timepoint for TFH1 and TFH2 and the pre-vaccination timepoint for TFD. We then calculated the $\hbox {TF}\Delta$ values by calculating the differences per transcript between TFH1 and TFH2 to obtain 7311 $\hbox {TF}\Delta$ time series (after removal of transcripts not overlapping across TFH1 and TFH2).

#### Total saliva proteomics

We profiled the total saliva proteome, using isobaric tandem mass tags (TMT) for quantitation using LC-MS/MS (liquid chromatography followed by mass spectrometry). We identified 12,473 proteins overall, with 4141 proteins (UniProt identifiers^38^) based on 2 unique peptides per protein, (11,005 proteins based on 1 unique peptide per protein) overall across all 95 samples where proteomics was carried out. Relative protein intensities were computed against a common pooled sample comprising of multiple healthy (pre-vaccination) weekly samples that was used across all TMT sample pools. The data were thus combined, and normalized using a Box-Cox^39^ transformation to obtain normal distributions. To construct the time series, the data were filtered again for 2 unique peptides, having less than 1/4 missing values, and no constant time series to obtain 724, 956, 759, and 662 proteomics time series for TFH1, TFH2, $\hbox {TF}\Delta$ and TFD respectively. All timepoint intensities were defined with respect to the first timepoint intensity for the hourly series for each respective protein, and to the vaccination day for TFD.

### Analysis of saliva extracellular vesicles

**Figure 2:**
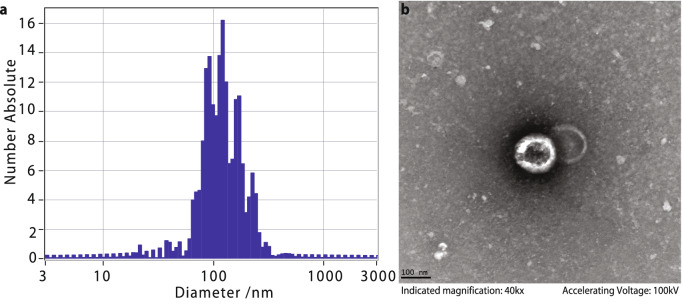
Extracellular vesicle profiles. (**a**) The EV size was profiled using ZetaView (Particle Metrix) with median concentrations of $6.2\times 10^{10}$ particles/ml with EV peak of $114.5\pm 4\,\hbox { nm}$. (**b**) EVs were imaged using transmission electron microscopy.

In addition to considering total saliva, we also implemented consistent extraction of EVs from 1 ml saliva, using ExoQuick-TC (SBI) and an overnight precipitation to obtain EV pellets, from which we extracted RNA. We carried out nanoparticle tracking analysis (ZetaView, Particle Metrix) and recorded median concentrations of $6.2\times 10^{10}$ particles/ml with EV peak of $114.5\pm 4\hbox { nm}$ (Fig. 2).

**Figure 3:**
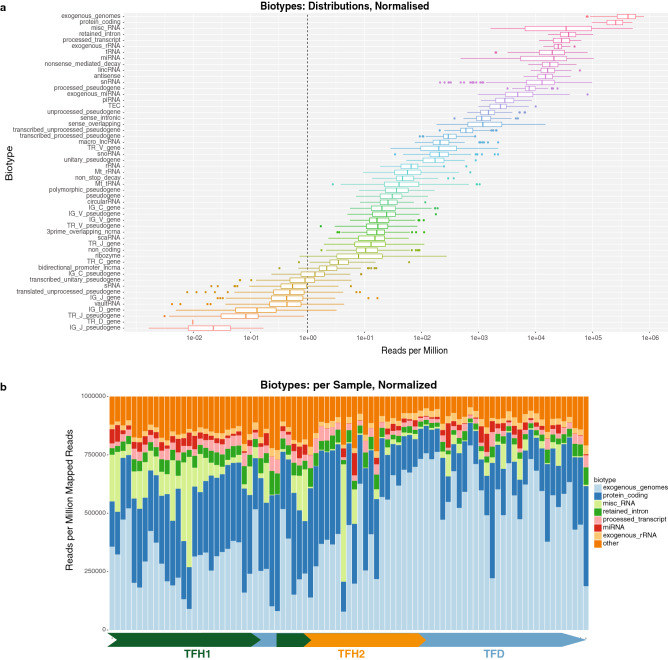
Extracellular vesicle biotypes. (**a**) Various biotypes were detected in EVs, with the overall distributions as shown (results from exceRpt^40^ mapped small-RNA-seq from saliva, $\sim$ 7 to 20 $\hbox {M}$ reads/sample, 50 bp/read). (**b**) The distributions of hourly and daily samples showed variability, particularly in the reduced exogenous genome content in the hourly samples.

The extracted EV RNA was sequenced (small RNA-sequencing) and the results were mapped to multiple databases using the exceRpt^40^ through http://genboree.org^41^. These included GENCODE transcripts, PIWI interacting RNA (piRNA), micro RNA (miRNA), and exogenous genomes and exogenous ribosomal RNA (rRNA) genomes, and multiple other biotypes (Fig. 3). The various biotypes detected have different variabilities (Fig. 3a). The majority of detected reads are from exogenous genomes ($\sim 10^6$) and protein coding (GENCODE transcripts, $\sim 10^6$ as well). The different biotypes per sample (Fig. 3b), for the TFH1, TFH2 and TFD time frames are indicative of a change in the relative distributions of the biotypes for the daily samples following the vaccination (increase of exogenous genomes content). This may be partly attributed to sampling as all TFD samples were taken at 8 am, and the corresponding early samples for TFH1 and TFH2 are also similar in biotype relative abundances, but differ in later samples during each day.

**Figure 4:**
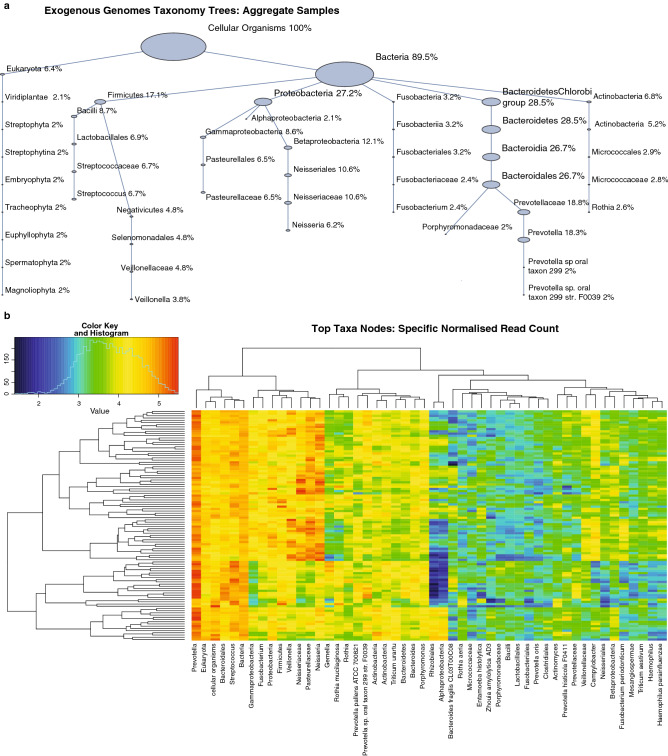
Exogenous taxa from extracellular vesicle analysis. (**a**) The exogenous genomes taxonomy tree based on mapped EV reads from the aggregate samples is shown. The tree has been truncated to include only nodes with relative reads compared to the Cellular Organisms root of $\ge 2\%$. (**b**) The top taxa nodes indicate consistency across samples (vertical axis).

In terms of the exogenous genomes, taxonomy trees were constructed per sample, and also for the aggregate samples using Genboree^42^ (Fig. 4a). The majority of abundances were assigned to bacteria (89.5%), and to Eukaryota (6.4%), where in terms of majority assignments at the next level, Bacteroidetes/Chlorobi group (28.5%), 27.2% were assigned to Proteobacteria and 17.1% to Firmicutes. Clustering of the overall top taxa by normalized read count was indicative of consistency across samples, with no significant sub-grouping at this level (Fig. 4b).

We again carried out downstream analysis in MathIOmica^37^, to create EV time series for mapped data for each of the three time frames (TFH1, TFH2, TFD), and the paired difference ($\hbox {TF}\Delta$) for GENCODE mapped entities, host piRNA, host miRNA and exogenous rRNA and exogenous taxa. Time series were constructed for entities where 0 count values were tagged as Missing, counts $< 1$ were considered equivalent noise, transcripts with more than 1/4 timepoints missing were removed, and transcripts with constant values across all timepoints were also removed.

**Table 1:** Time series counts and classifications across saliva omics for the various time frames.

Time frames	mRNA	protein	EV GENCODE	EV piRNA	EV miRNA	EV taxa	EV rRNA taxa	Total
**A. TFH1**								
Total series	15,621	724	55,971	589	258	353	1015	74,531
Total classed	5781	671	27,684	434	113	409	280	35,372
Lag 1	1085	664	5328	145	84	224	125	7655
Spike Max	6	6	7767	72	7	0	1	7859
Spike Min	3597	0	11,328	23	14	148	18	15,128
Other lags	1093	1	3261	194	8	37	136	4730
**B. TFH2**								
Total series	7493	956	55,499	312	142	218	711	65,331
Total classed	2707	252	21,981	224	63	328	184	25,739
Lag 1	481	164	8638	104	29	232	126	9774
Spike Max	2	1	5762	31	2	0	0	5798
Spike Min	1685	2	4926	19	24	69	10	6735
Other lags	539	85	2655	70	8	27	48	3432
$$\mathbf{C}. TF \Delta$$								
Total series	7311	759	58,596	275	140	212	682	67,975
Total classed	1200	441	16,517	191	60	227	179	18,815
Lag 1	369	433	6490	91	56	169	95	7703
Spike Max	3	1	2947	6	0	0	0	2957
Spike Min	2	4	2427	0	0	2	0	2435
Other lags	826	3	4653	94	4	56	84	5720
**D. TFD**								
Total series	8155	662	58,863	354	218	307	841	69,400
Total classed	3762	169	22,146	296	30	418	273	27,094
Lag 1	439	38	5567	92	1	180	126	6443
Spike Max	2	3	5271	18	0	0	0	5294
Spike Min	2248	1	5953	7	11	165	11	8396
Other lags	1073	127	5355	179	18	73	136	6961

### Temporal trends identified in saliva

#### Time series classification

The time series for all omics discussed below were classified into temporal trends using MathIOmica’s^37^ spectral methods that classify signals based on their autocorrelations, i.e. correlating a time signal with a delayed version of itself, where the delay is characterized as a time *lag* (e.g. lag 1 corresponds to a delay of 1 time interval unit). The method uses a Lomb–Scargle transformation^43–45^ to generate periodograms whose inverse Fourier transform can then produce a set of autocorrelations at different lags for a given time series. Our classification successively identifies time series from the dataset that have statistically significant autocorrelations at particular time lags. In summary (see “Methods”), MathIOmica’s classification generates three sets of classes, strictly based on temporal behavior: (1) significant autocorrelations at various lags, (2) no autocorrelations, but with positive spikes (abnormally high signals above baselines present at single timepoints), (3) no autocorrelations, but negative spikes present (abnormally low signals below baseline at single timepoints). Within each class a two-tier classification into groups and subgroups is carried out: this approach first separates within-class autocorrelation groups by clustering on autocorrelation lags: signals that may have statistically significant autocorrelation for the class lag, but may still exhibit underlying different structure at other lags. Additionally, the second level clustering into subgroups is based on intensities, and allows us to separate signals that may have different phase (directionality/sense), which cannot be obtained from the periodograms.

The analysis was carried out for each of the omics individually and thousands of individual component trends were identified in the different classes and subgroupings therein. A brief summary is provided in Table 1. The entirety of visualizations and classification memberships are available in the online data files (ODFs), including heatmaps per omic per individual time frame trends, as well as all the code to generate these. We also combined all the classified information to obtain an integrated view of the various omics. Below we showcase parts of the mRNA analysis, as well the results of all the omics combined.

#### Saliva mRNA data analysis

**Figure 5:**
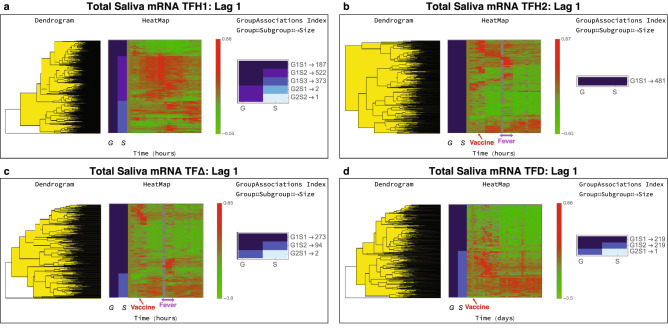
Total saliva mRNA time series trends. The Lag 1 classification results from MathIOmica are shown for the different time frames. (**a**) During TFH1 the subject followed their normal routine. (**b**) During TFH2, the subject was vaccinated with PPSV23. For both TFH1 and TFH2 the first timepoint corresponds to 7 am. Vaccination took place at 10.30 am. The subject reported fever from 5.30 to 10 pm. (**c**) The $\hbox {TF}\Delta$ results correspond to paired differences between TFH2 and TF1 hourly points, to remove intra-day variation so as to focus on the perturbation vaccination responses. The plot is indicative of a response to the vaccine and a response that coincides with the reported fever. (**d**) For the daily data, TFD, the corresponding vaccine timepoint is Day 3. There is a direct response the days following the vaccination, and a different response in a subset of genes approximately a week following the vaccination, corresponding to immune system activation.

The trends shown in Fig. 5 correspond to the mRNA time series showing statistically significant time series trends (p value $< 0.01$ based on bootstrap simulations, n = 100,000) for each of the time frames, for Lag 1 classes.

**Table 2:** Statistically significant reactome^46^ pathways identified ($\hbox {FDR}< 3\times 10^{-3}$ results shown).

Reactome pathway	Matched IDs	p value	FDR
**A. Total saliva mRNA:** $\mathbf{TF} \Delta$			
(i) Class: Lag 1, G1S1			
Endosomal/vacuolar pathway	15	$1.6\times 10^{-9}$	$1.6\times 10^{-6}$
Antigen presentation: folding, assembly and peptide loading of class I MHC	16	$4.0\times 10^{-9}$	$2.0\times 10^{-6}$
Interferon gamma signaling	22	$1.8\times 10^{-7}$	$5.9\times 10^{-5}$
ER-Phagosome pathway	17	$5.0\times 10^{-7}$	$1.1\times 10^{-4}$
Interferon alpha/beta signaling	18	$5.7\times 10^{-7}$	$1.1\times 10^{-4}$
Antigen processing-cross presentation	17	$2.7\times 10^{-6}$	$4.4\times 10^{-4}$
Insulin-like growth factor-2 mRNA binding proteins (IGF2BPs/IMPs/VICKZs) bind RNA	5	$1.6\times 10^{-5}$	$2.2\times 10^{-3}$
Attenuation phase	8	$1.8\times 10^{-5}$	$2.2\times 10^{-3}$
Interferon signaling	24	$2.5\times 10^{-5}$	$2.8\times 10^{-3}$
Neutrophil degranulation	27	$2.8\times 10^{-5}$	$2.8\times 10^{-3}$
(ii) Class: Lag 1, G1S2			
Translocation of ZAP-70 to Immunological synapse	14	$1.1\times 10^{-16}$	$2.2\times 10^{-14}$
Phosphorylation of CD3 and TCR zeta chains	14	$1.1\times 10^{-16}$	$2.2\times 10^{-14}$
Generation of second messenger molecules	15	$1.1\times 10^{-16}$	$2.2\times 10^{-14}$
PD-1 signaling	14	$1.1\times 10^{-16}$	$2.2\times 10^{-14}$
Interferon gamma signaling	22	$1.8\times 10^{-15}$	$2.8\times 10^{-13}$
Costimulation by the CD28 family	15	$2.4\times 10^{-14}$	$3.1\times 10^{-12}$
TCR signaling	17	$4.2\times 10^{-14}$	$4.7\times 10^{-12}$
Downstream TCR signaling	16	$4.8\times 10^{-14}$	$4.7\times 10^{-12}$
Interferon signaling	23	$1.7\times 10^{-12}$	$1.5\times 10^{-10}$
MHC class II antigen presentation	14	$1.1\times 10^{-10}$	$8.8\times 10^{-9}$
Cytokine signaling in immune system	33	$5.5\times 10^{-8}$	$3.9\times 10^{-6}$
Insulin-like growth factor-2 mRNA Binding Proteins (IGF2BPs/IMPs/VICKZs) bind RNA	5	$1.6\times 10^{-7}$	$1.0\times 10^{-5}$
Immune system	50	$1.1\times 10^{-6}$	$6.4\times 10^{-5}$
Neutrophil degranulation	16	$8.2\times 10^{-6}$	$4.6\times 10^{-4}$
Adaptive immune system	23	$3.3\times 10^{-5}$	$1.7\times 10^{-3}$
**B. Total saliva mRNA: TFD**			
(i) Class: Lag 1, G1S1			
Endosomal/vacuolar pathway	16	$4.1\times 10^{-11}$	$3.6\times 10^{-8}$
Interferon alpha/beta signaling	22	$1.3\times 10^{-10}$	$5.7\times 10^{-8}$
Interferon signaling	29	$8.9\times 10^{-9}$	$2.6\times 10^{-6}$
Cytokine signaling in immune system	59	$2.8\times 10^{-9}$	$4.6\times 10^{-6}$
Interferon gamma signaling	22	$3.1\times 10^{-8}$	$5.3\times 10^{-6}$
Antigen presentation: folding, assembly and peptide loading of class I MHC	14	$5.4\times 10^{-8}$	$7.8\times 10^{-6}$
Insulin-like growth factor-2 mRNA Binding Proteins (IGF2BPs/IMPs/VICKZs) bind RNA	6	$4.3\times 10^{-7}$	$4.8\times 10^{-5}$
Immune system	97	$4.4\times 10^{-7}$	$4.8\times 10^{-5}$
Antigen processing-cross presentation	17	$6.7\times 10^{-7}$	$6.5\times 10^{-5}$
Interleukin-4 and interleukin-13 signaling	18	$7.8\times 10^{-7}$	$6.7\times 10^{-5}$
ER-phagosome pathway	14	$1.4\times 10^{-5}$	$1.1\times 10^{-3}$
(ii) Class: Lag 1, G1S2			
Response of EIF2AK4 (GCN2) to amino acid deficiency	13	$3.6\times 10^{-7}$	$1.5\times 10^{-4}$
Cytokine signaling in immune system	51	$4.1\times 10^{-7}$	$1.5\times 10^{-4}$
Immune system	88	$4.9\times 10^{-7}$	$1.5\times 10^{-4}$
ATF4 activates genes in response to endoplasmic reticulum stress	7	$4.4\times 10^{-6}$	$9.8\times 10^{-4}$
Innate immune system	48	$7.1\times 10^{-6}$	$1.3\times 10^{-3}$
PERK regulates gene expression	7	$1.7\times 10^{-5}$	$2.0\times 10^{-3}$
CLEC7A/inflammasome pathway	4	$1.8\times 10^{-5}$	$2.0\times 10^{-3}$
Interleukin-1 processing	4	$1.8\times 10^{-5}$	$2.0\times 10^{-3}$

##### Hourly results (TFH1, TFH2 and ${TF}\Delta$)

The saliva mRNA showed variation across the day in the untreated TFH1 period. Overall 5781 time series of mRNA isoforms were found to have statistically significant trends, with 1085 Lag 1, 6 Spike Max, 3597 Spike Min and 1093 other Lag class memberships. The Lag 1 group is shown in Fig. 5a, where the 1085 time series are further assigned into groups and subgroups based on clustering, according to their different temporal behaviors as described above. In the Lag 1 classification in Fig. 5a there are two groups (G1 and G2). G1 has 3 subgroups (S), S1, S2 and S3 with 187, 522 and 373 time series in each respectively. G2 has 2 subgroups S1, S2 with two and one time series respectively. The groupings show substantial variation in these isoforms’ intensities during the day, with G1S1 showing gradual decreases, G1S2 peaking after morning until the evening, and G1S3 showing peaks later in the evening and night (the first timepoint is at 7 am).

In the analysis of the 24 h period spanning vaccination, TFH2, we should note that the subject reported fever $\sim$ 7 h post vaccination, lasting for about 4.5 h. The classification identified 2707 isoform time series, with 481 in Lag 1, 2 in Spike Max, 1685 in Spike Min, and 539 in other Lag ($\ge 2$) classes. The clustering results for Lag 1 are also shown in Fig. 5b. The changes are indicative of the activation response due to the vaccination.

Given the variation observed in TFH1, we constructed the $\hbox {TFH}\Delta$ time series, using paired differences of intensities at each timepoint. The approach aimed to remove non-vaccination daily variation, and resulted in 1200 time series with statistically significant trends. These included 369 Lag 1, 3 Spike Max, 2 Spike Min, and 826 other Lags memberships. The Lag 1 results are shown in Fig. 5c. The subgroupings of 273 G1S1 isoform time series, show punctuated trends following vaccination and also coincidental with the reported fever, lasting about 4 h (timepoints). Furthermore, the G1S2 subgroup of 94 time series is indicative of up-regulation following the vaccination. Additionally a distinct up-regulation of a subset of genes is observed to coincide with the reported fever (Fig. 5c).

We carried out Gene Ontology (GO)^47^ and Reactome Pathway enrichment analysis^46,48^ and identified multiple involved pathways. Results for $\hbox {TF}\Delta$ with False Discovery Rate, FDR $<3\times 10^{-3}$ are shown in Table 2, and full results available in the ODFs. For the set of $\hbox {TF}\Delta$ genes showing immediate response post vaccination and response during the fever period (Class Lag 1, G1S1 Fig. 5c), results include (Table 2A (i)) endosomal/vacuolar pathway, antigen presentation (class 1 MHC) and processing, interferon gamma and alpha/beta signaling, neutrophil degranulation and ER-Phagosome pathways indicative of the immune activation. Furthermore, a set of genes that show continued up-regulation following the vaccination (Class Lag 1, G1S2 Fig. 5c) had enrichment of various immunological pathways including TCR signaling-related pathways, PD-1 signaling, also Interferon gamma signaling, Costimulation by the CD28 family, MHC class II antigen presentation, Cytokine Signaling in Immune system, and also Neutrophil degranulation pathways and general Immune System pathways.

##### Daily results (TFD)

We also monitored the daily changes post vaccination for 1 month. For the mRNA analysis, 3762 time series had statistically significant trends. These included 439 Lag 1, 2 Spike Max, 2248 Spike Min, and 1073 other Lag memberships. The Lag 1 TFD results are shown in Fig. 5d. As shown in the figure, in the G1S1 subgroup 219 isoform time series show up-regulation, for about 11 days post vaccination, followed by a return to lower expression levels. The 218 time series in the G1S2 subgroup also show a later up-regulation response, after 11 days compared to G1S1, again following the vaccination, and lasting for the remainder of the daily observation period.

Reactome pathway and GO enrichment analysis also identified multiple pathways corresponding to each trend. Reactome results for TFD Lag 1 are shown in Table 2B(i)–(ii) for Lag 1 G1S1 and G1S2 subgroups (full results, including GO terms available in the ODFs). In the TFD Lag 1 G1S1 group, over-representation included Endosomal/Vacuolar pathway (16 genes) Interferon Signaling (29 genes), Cytokine Signaling in Immune system (59 genes), Antigen Presentation (MHC I related, 14 genes) and inteleukin 4 and 13 signaling pathways (18 genes), and Immune System (97 genes). These are indicative of an early response within the first days after vaccination. For TFD Lag 1 G1S2 the results included general Immune System activation (88 genes), and also Cytokine Signaling in Immune System (51 genes) as pathways with statically significant over-representation and having the most genes identified.

### Other omics

In addition to the mRNA, each other set of omics was individualized analyzed to identify temporal trends, using the same classification method in MathIOmica as described above. This identified statistically significant trends for time series for different omics in the different time frames are shown in Table 1.

**Figure 6:**
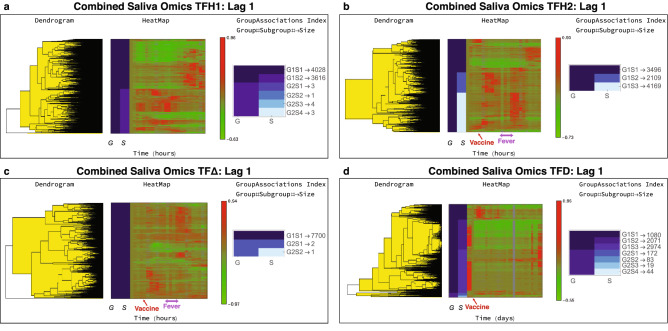
Combined saliva omics time series trends. The Lag 1 classification results from MathIOmica are shown for the different time frames. (**a**) During TFH1 the subject followed their normal routine. (**b**) During TFH2, the subject was vaccinated with PPSV23. For both TFH1 and TFH2 the first timepoint corresponds to 7 am. Vaccination took place at 10.30 am. The subject reported fever from 5.30 to 10 pm. (**c**) The $\hbox {TF}\Delta$ results correspond to paired differences between TFH2 and TFH1 hourly points, to remove intra-day variation so as to focus on the perturbation vaccination responses. The plot is indicative of a phased response to the vaccine compared to the mRNA responses and a response that is again shifted compared to the reported fever (cf. Fig. 5c). (**d**) For the daily data, TFD, the corresponding vaccine timepoint is Day 3. There is a direct response the days following the vaccination. The EV omics dominate the information in this plot, compared to the mRNA in total saliva response (cf. Fig. 5d).

The different classes for the omics datasets were joined within each respective time frame, and data within each combined class were clustered together. The breakdown of identified trends included overall 35,372 time series for TFH1, 25,739 for TFH2, 18,815 for $\hbox {TF}\Delta$, and 27,094 for TFD. In reference to the corresponding omics, the EV GENCODE identifiers accounted for more that 78% of the time series across all time frames. The results from Lag 1 are again shown in Fig. 6. In terms of temporal behavior, we notice again similar responses in the various time frames. In TFH1, we again see large temporal variation, with sets of time series being up-regulated during awake time and a subset during night time as shown in Fig. 6a. In TFH2 (Fig. 6b), the effects of the vaccination become apparent with up-regulated responses following the vaccination.

In the paired comparison $\hbox {TF}\Delta$ for Lag 1 in Fig. 6c, there is a major subgroup (G1S1) with 7700 time series. A subset of the omics show increases in intensity about 2–3 h post vaccination for a few hours, and additionally again increase in intensity about 14 h post vaccination (after the fever time span). We note here that the response/time pattern appear lagging compared to the corresponding mRNA results in Fig. 5c, by approximately 3 h, both following the immediate vaccine response, and also for the reported fever time span. The mRNA over-representation analysis was discussed above. Proteomics displayed similar trends to the mRNA. For Lag 1 the Reactome analysis for the aggregate group/subgroup proteins resulted in multiple statistically significant pathways ($FDR < 0.01$), where the top pathways (FDR $<5.9\times 10^{-13}$) included Neutrophil degranulation (96 entities identified), Innate Immune System (135 entities), Immune System (with 158 entities identified), and more (the top 20 ranked by smallest FDR are shown in Table 3A, $\hbox {FDR} < 2.6\times 10^{-7}$).

GO analysis for the EV GENCODE results for $\hbox {TF}\Delta$ Lag 1 were generic and included multiple cellular processes. The results appear as non-specific to immune response, particularly as no over-representation in Reactome pathways was found to be statistically significant based on FDR. For EV miRNA the $\hbox {TF}\Delta$ Lag 1 showed statistically significant over-representation in GTP binding (p value $<4.6\times 10^{-14}$), perinuclear region of cytoplasm (p value $<9.3\times {10^{-14}}$), nerve growth factor receptor signaling pathway (p alue $<1.5\times 10^{-13}$), MAPK cascade (p value $<1.7\times 10^{-13}$), MYD88 dependent toll-like receptor signaling pathway (p value $<1.7\times 10^{-13}$), toll-like receptor signaling pathway (p value $< 3.4\times 10^{-13}$) and others.

Furthermore, in the TFD combined omics results, there are three main trends (Fig. 5d), with: (1) G1S1 showing 1080 series showing a relative increase in intensity in the days after immunization, and decrease to pre-immunization levels after about 12 day. (2) G1S2 with 2071 series showing a decrease in intensity, with some return to pre-immunization levels after about one month. (3) G1S3 with 2974 components showing a set of time series that decrease in intensity, including some that return to post vaccination levels and some that remain at lower levels, and a set that remains constant in intensity for the duration of the month’s measurements and increases towards the end of the monthly period. The mRNA TFD pathway analysis results were discussed above (Table 2B). Proteomics time series on the other hand displayed fewer pathway enrichment results as shown in Table 3B, including various Nonsense-Mediated Decay pathways, Translation related pathways, ROBO receptor signaling and others. GO analysis for the EV GENCODE results for TFD Lag 1 included generic molecular functions such as protein binding and ATP binding and biological processes relating to protein phosphorylation (105 IDs, adjusted p value $< 4.7\times 10^{-5}$). Full enrichment analysis results for all classifications and omics are available in the ODFs on Zenodo.

**Table 3:** Reactome pathway enrichment analysis results for protein Lag 1 aggregate results for $\hbox {TF}\Delta$ and TFD (top 20 pathways based on FDR for each time frame shown).

Reactome pathway	Matched IDs	p value	FDR
**A. Total saliva protein: TF**$\Delta$			
Neutrophil degranulation	96	$1.1\times 10^{-16}$	$6.8\times 10^{-14}$
Innate immune system	135	$1.1\times 10^{-16}$	$6.8\times 10^{-14}$
Immune system	158	$1.4\times 10^{-15}$	$5.9\times 10^{-13}$
Signaling by ROBO receptors	34	$1.2\times 10^{-13}$	$3.8\times 10^{-11}$
Regulation of expression of SLITs and ROBOs	29	$9.5\times 10^{-13}$	$2.3\times 10^{-10}$
Eukaryotic translation elongation	22	$1.7\times 10^{-12}$	$3.5\times 10^{-10}$
Cellular responses to stress	58	$3.3\times 10^{-12}$	$5.7\times 10^{-10}$
Cellular responses to external stimuli	58	$9.0\times 10^{-12}$	$1.4\times 10^{-9}$
Peptide chain elongation	20	$3.8\times 10^{-11}$	$5.2\times 10^{-9}$
Nonsense-mediated decay (NMD)	22	$6.8\times 10^{-11}$	$7.5\times 10^{-9}$
Nonsense mediated decay (NMD) enhanced by the Exon Junction Complex (EJC)	22	$6.8\times 10^{-11}$	$7.5\times 10^{-9}$
Nonsense mediated decay (NMD) independent of the exon junction complex (EJC)	20	$7.7\times 10^{-11}$	$7.8\times 10^{-9}$
Eukaryotic translation termination	20	$1.8\times 10^{-10}$	$1.7\times 10^{-8}$
SRP-dependent cotranslational protein targeting to membrane	21	$2.0\times 10^{-10}$	$1.8\times 10^{-8}$
Viral mRNA translation	20	$6.1\times 10^{-10}$	$4.9\times 10^{-8}$
Formation of a pool of free 40S subunits	19	$1.2\times 10^{-9}$	$8.8\times 10^{-8}$
Platelet degranulation	21	$2.4\times 10^{-9}$	$1.7\times 10^{-7}$
Selenocysteine synthesis	19	$3.2\times 10^{-9}$	$2.2\times 10^{-7}$
Regulation of insulin-like growth factor (IGF) transport and uptake by insulin-like growth factor binding proteins (IGFBPs)	20	$3.7\times 10^{-9}$	$2.4\times 10^{-7}$
Response of EIF2AK4 (GCN2) to amino acid deficiency	19	$4.3\times 10^{-9}$	$2.6\times 10^{-7}$
**B. Total saliva protein: TFD**			
Eukaryotic translation elongation	6	$3.5\times 10^{-7}$	$1.6\times 10^{-4}$
Nonsense-mediated decay (NMD)	6	$1.1\times 10^{-6}$	$1.6\times 10^{-4}$
Nonsense mediated decay (NMD) enhanced by the exon junction complex (EJC)	6	$1.1\times 10^{-6}$	$1.6\times 10^{-4}$
Signaling by ROBO receptors	7	$3.2\times 10^{-6}$	$2.9\times 10^{-4}$
Translation	8	$3.2\times 10^{-6}$	$2.9\times 10^{-4}$
Peptide chain elongation	5	$6.9\times 10^{-6}$	$4.7\times 10^{-4}$
Nonsense mediated decay (NMD) independent of the exon junction complex (EJC)	5	$8.3\times 10^{-6}$	$4.7\times 10^{-4}$
Regulation of expression of SLITs and ROBOs	6	$1.0\times 10^{-5}$	$4.7\times 10^{-4}$
Formation of a pool of free 40S subunits	5	$1.1\times 10^{-5}$	$4.7\times 10^{-4}$
Eukaryotic translation termination	5	$1.1\times 10^{-5}$	$4.7\times 10^{-4}$
Selenocysteine synthesis	5	$1.4\times 10^{-5}$	$4.9\times 10^{-4}$
Viral mRNA translation	5	$1.5\times 10^{-5}$	$4.9\times 10^{-4}$
Interleukin-6 signaling	3	$1.5\times 10^{-5}$	$4.9\times 10^{-4}$
Response of EIF2AK4 (GCN2) to amino acid deficiency	5	$1.6\times 10^{-5}$	$4.9\times 10^{-4}$
SRP-dependent cotranslational protein targeting to membrane	5	$1.8\times 10^{-5}$	$4.9\times 10^{-4}$
GTP hydrolysis and joining of the 60S ribosomal subunit	5	$1.9\times 10^{-5}$	$4.9\times 10^{-4}$
L13a-mediated translational silencing of ceruloplasmin expression	5	$1.9\times 10^{-5}$	$4.9\times 10^{-4}$
Eukaryotic translation initiation	5	$2.8\times 10^{-5}$	$6.4\times 10^{-4}$
Cap-dependent translation initiation	5	$2.8\times 10^{-5}$	$6.4\times 10^{-4}$
Interleukin 6-family signaling	3	$8.0\times 10^{-5}$	$1.8\times 10^{-3}$

To identify a set of time series omics that showed statistically significant temporal trends (adjusted p value $<0.01$) in multiple time frames, we intersected the results for Lag 1 for the various omics for Lag 1 $\hbox {TF}\Delta$ and TFD time frames. The overlaps for the omics considered included 43 mRNA, 16 protein, 658 EV GENCODE, 30 EV piRNA, 41 EV exogenous taxa, and 45 EV rRNA taxa time series (mRNA, protein, piRNA and exogenous taxa memberships are shown in Table 4). We carried out Reactome pathway analysis on the overlaps directly. For mRNA we observed over-representation in Insulin-like Growth Factor-2 mRNA Binding Proteins (IGF2BPs/IMPs/VICKZs) bind RNA (FDR $< 1.6\times 10^{-6}$), CLEC7A/inflammasome pathway (FDR $<1.3\times 10^{-3}$), Interleukin-4 and Interleukin-13 signaling (FDR $< 0.043$)—the genes involved include CD44 and IL1B, that are associated with monocyte aggregation, and FCGR2B and SAMSN1 that are involved in negative regulation of B-cell proliferation. Other omics overlaps did not show statistically significant over-representations. We note that the most represented pathway for the protein overlaps is Immune System, with 7 entities (RAB27A, ATP6V1B2, PPP2R1A, VASP, B4GALT1, TLN1, VCL; p value $< 0.024$, FDR $< 0.072$).

**Table 4:** Overlaps of responding components between time frames $\hbox {TF}\Delta$ and TFD.

Omics component	TF$\Delta \cap$TFD Lag 1 time series
mRNA total saliva*	ADGRG3, AIF1, B2M, CD44, CIR1, EDEM1, ETS2, FBP1, FCGR2B, FOSL2, GBP1, GLUL, GRB2, H3F3A, IL1B, INHBA, ITGAM, KIAA0226L, KIF1C, LAMC2, MLLT6, MMP12, MYL12B, PGS1, PLEK, PLEKHM1P, PPL, PREX1, PTPRC, RGS2, RP11-463O12.5, RP11-482G13.1, RPL26, S100A8, SAMSN1, SCARF1, SERINC5, SLC25A37, TAGAP, TMEM41A, TMSB10, TMSB4X, VCAN
Proteomics total saliva*	ARF4, ATP6V1B2, B4GALT1, EEF1B2, H2AFY, NACA, OLA1, PPP2R1A, RAB27A, RPL6, RTN3, SAA2-SAA4, TLN1, TTN, VASP, VCL
EV piRNA	hsa_piR_000421, hsa_piR_000999, hsa_piR_001790, hsa_piR_003329, hsa_piR_004220, hsa_piR_004341, hsa_piR_004413, hsa_piR_006415, hsa_piR_007567, hsa_piR_007776, hsa_piR_008631, hsa_piR_009273, hsa_piR_009330, hsa_piR_009903, hsa_piR_010461, hsa_piR_010687, hsa_piR_011519, hsa_piR_012576, hsa_piR_012637, hsa_piR_013314, hsa_piR_014751, hsa_piR_016236, hsa_piR_016960, hsa_piR_017791, hsa_piR_017961, hsa_piR_019030, hsa_piR_019478, hsa_piR_020493, hsa_piR_020727, hsa_piR_021487
EV exogenous taxa	*Acyrthosiphon pisum, Aerococcaceae, Aggregatibacter actinomycetemcomitans, Aggregatibacter aphrophilus F0387, Aggregatibacter segnis ATCC 33393, Aggregatibacter sp. oral taxon 458 str. W10330, Atopobium, Ecdysozoa, Haemophilus pittmaniae HK 85, Ixodes scapularis, Lachnospiraceae bacterium oral taxon 082 str. F0431, Leptotrichiaceae, Onchocerca volvulus, Phytophthora infestans, Porphyromonas sp. oral taxon 278 str. W7784, Prevotella melaninogenica, Prevotella melaninogenica ATCC 25845, Prevotella oris C735, Prevotella oulorum F0390, Prevotella sp. oral taxon 472 str. F0295, Prevotella veroralis F0319, Rhizobium, Streptococcaceae, Streptococcus gordonii str. Challis substr. CH1, Streptococcus mitis 17/34, Streptococcus mitis 18/56, Streptococcus mitis 29/42, Streptococcus mitis ATCC 6249, Streptococcus mitis SK321, Streptococcus mitis SK564, Streptococcus mitis SK569, Streptococcus mitis SK575, Streptococcus pneumoniae, Streptococcus pneumoniae GA47179, Streptococcus pseudopneumoniae, Streptococcus sp. M334, Strigamia maritima, Triticeae,* unclassified *Lachnospiraceae, Veillonella sp. 3_1_44, Veillonella sp. oral taxon 158 str. F0412*

* Official Gene Names: *ADGRG3* adhesion G protein-coupled receptor G3, *AIF1* allograft inflammatory factor 1, *ARF4* ADP ribosylation factor 4, *ATP6V1B2* ATPase H+ transporting V1 subunit B2, *B2M* beta-2-microglobulin, *B4GALT1* beta-1,4-galactosyltransferase 1, *CD44* CD44 molecule, *CIR1* corepressor interacting with RBPJ, *1, EDEM1* ER degradation enhancing alpha-mannosidase like protein 1, *EEF1B2* eukaryotic translation elongation factor 1 beta 2, *ETS2* ETS proto-oncogene 2, transcription factor, *FBP1* fructose-bisphosphatase 1, *FCGR2B* Fc fragment of IgG receptor IIb, *FOSL2* FOS like 2, AP-1 transcription factor subunit, *GBP1* guanylate binding protein 1, *GLUL* glutamate-ammonia ligase, *GRB2* growth factor receptor bound protein 2, *H2AFY* H2A histone family member Y, *H3F3A* H3 histone family member 3A, *IL1B* interleukin 1 beta, *INHBA* inhibin beta A subunit, *ITGAM* integrin subunit alpha M, *KIF1C* kinesin family member 1C, *LAMC2* laminin subunit gamma 2, *MLLT6* MLLT6, PHD finger domain containing, *MMP12* matrix metallopeptidase 12, *MYL12B* myosin light chain 12B, *NACA* nascent polypeptide-associated complex alpha subunit, *OLA1* Obg like ATPase 1, *PGS1* phosphatidylglycerophosphate synthase 1, *PLEK* pleckstrin, *PLEKHM1P* Pleckstrin Homology And RUN Domain Containing M1 Pseudogene 1 2 3 5, *PPL* periplakin, *PPP2R1A* protein phosphatase 2 scaffold subunit Aalpha, *PREX1* phosphatidylinositol-3,4,5-trisphosphate dependent Rac exchange factor 1, *PTPRC* protein tyrosine phosphatase, receptor type C, *RAB27A* RAB27A, member RAS oncogene family, *RGS2* regulator of G-protein signaling 2, *RP11-463O12.5* to be experimentally confirmed, *RP11-482G13.1* novel transcript similar to family member with sequence similarity 157, *RPL26* ribosomal protein L26, *RPL6* ribosomal protein L6, *RTN3* reticulon 3, *S100A8* S100 calcium binding protein A8, *SAA2–SAA4* SAA2–SAA4 readthrough, *SAMSN1* SAM domain, SH3 domain and nuclear localization signals 1, *SCARF1* scavenger receptor class F member 1, *SERINC5* serine incorporator 5, *SLC25A37* solute carrier family 25 member 37, *TAGAP* T-cell activation RhoGTPase activating protein, *TLN1* talin 1, *TMEM41A* transmembrane protein 41A, *TMSB10* thymosin beta 10, *TMSB4X* thymosin beta 4, X-linked, *TTN* titin, *VASP* vasodilator-stimulated phosphoprotein, *VCAN* versican, *VCL* vinculin.

Finally, for each class of the combined classified data, we constructed putative interaction networks based on the Euclidean distance between the class members. Here, we assumed that the omics showing similar trends over time within each class are likely to be associated to each other (even though interactions may be indirect). The group/subgroup annotations within each class were also included. The constructed networks are available as a resource in the ODFs, including *.json* files, and may be used to explore and validate possible interactions in saliva data (see online Methods).

## Discussion

We have presented here our findings from a case study of the utility of saliva towards personalized health monitoring. Following vaccination of a subject with pneumococcal vaccine (PPSV23) we were able to detect distinct signatures in various saliva omics. We were able to profile more than 65,000 components in various time frames over time, and identify 18,000+ time series that had statistically significant temporal trends. The time series trends observed were indicative of immune response, which coincided in timing with the vaccination, and fever reported by our subject. The time frames of immune responses observed are concordant with our expectations of innate and adaptive immunity development, as seen both in the immediate hourly as well as the short- and long-term daily responses observed. Various pathways were activated, involved in immune response and regulation, including interferon signaling and MHC antigen presentation. The immune activation spanned an initial response within hours, as well as long term response extending for over a month.

Our results suggest that saliva omics can be consistently assessed for personalized monitoring. While multiple omics provide responses post-vaccination as discussed for the $\hbox {TF}\Delta$ and TFD time frames, mRNA results appear more specific and sensitive (timewise) to the vaccination. EV response, particularly transcript-level (GENCODE), though very sensitive and responsive does not yield as specific results as the mRNA from total saliva in a non-targeted approach. In fact many EV mRNAs show statistically significant temporal responses, which is likely due to increased EV release from various cells, but not specific in terms of reflecting the functional responses in the cells of origin. EV results also suggest a lagged response by a few hours compared to the mRNA observations, which also suggests that the mRNA measurements can potentially provide more timely data for practical health monitoring. Additional omics showed responses concordant with the mRNA responses, including miRNA, piRNA and exogenous taxa quantitations, however these need further validation, particularly as our knowledgebase of pathway implications and functional important of such omics is still under development. Some of the omics time series are found in responses across hourly and daily samplings (Table 4), and such sets of omics can be targeted for non-invasive health monitoring. The processing of samples for mRNA involved the use of standardized kits that can be used by subjects remotely, and can facilitate storage of samples for about a month without refrigeration. Additionally, the mRNA sequencing preparation and result analysis are considerably faster than the other omics processing, so our recommendation based on our findings is to utilize similar approaches for mRNA broad profiling, while using targeted protein/EV-content assays. These omics must be coupled with standard physiological and molecular measures already utilized in the clinic for a complete assessment of health status. While our results are based on PPSV23 activation, we anticipate that they can be extended to additional vaccine and immune disease profiling, particularly with the goal of discovering immune-specific signatures for each affliction and/or intervention.

Previous work on saliva omics has focused on the evaluation of omics as biomarkers, with less emphasis on temporal changes, including proteomic and transcriptomic evaluations, EV characterization, miRNA profiling and microbiome mapping^13–21,23–25,49,50^. With respect to the saliva proteome, Denny et al. first identified 1116 proteins^51^ using multidimensional separation. Yan et al. identified 1939 proteins from whole and ductal saliva compiled from multiple MS studies, in a comparison of saliva and plasma^52^. The coupling of hexapeptide libraries for dynamic range compression (DRC) with three-dimensional (3D) peptide fractionation by Bandhakavi et al., resulted in further protein identifications (2340 proteins)^53^. Such efforts were substantially improved by Grassl et al. who used deep profiling by mass spectrometry to identify more than 3700 human proteins^54^. Their study also assessed intra-day changes for waking and postprandial collection times, identifying enrichment of proteins associated with ‘antibiotic’ keywords in waking versus postprandial collection times. Our study significantly extends the temporal profiling aspect to hourly 24 h period monitoring (for 4141 proteins—UniProt IDs, with > 2 unique peptides used per identification), in addition to daily data for a month. We also observed substantial variability during the course of a day. To account for this daily variation, we used a paired two 24 h period comparison to elucidate changes particular to the vaccination (essentially subtracting normal hourly variation effects), and also limited collection to a single morning timepoint in our daily collection. Other studies of the saliva proteome have included the integration of transcriptomics with antibody-based proteomics^55–57^ to assess the salivary gland content (Human Protein Atlas), suggesting up to 15,218 proteins are expressed in the gland itself, though these studies did not dynamically profile secreted saliva.

Salivary proteomics (at various scales) has been used in prospective clinical applications ranging from periodontitis, oral and other cancers, diabetes, Sjogren’s syndrome, and to assess viral proteins in Zika virus, Dengue virus, HBV and HCV (review by Katsani and Sakellari^58^). Saliva immunoglobulins levels in COVID-19 were also evaluated by Isho et al.^59^. With respect to pneumonia, Klein Kremer et al. measured overall increases in aggregate salivary protein levels in children diagnosed with Lobar pneumonia^60^. Recently Tsai et al. reported immunoassay results on 9 cytokines and C-reactive protein (CRP), and detected higher levels of CRP and IL-6 in children with pneumonia^61^. In our MS-based study we did not detect CRP/IL-6 changes directly, but we identified multiple proteins associated with immune pathways (Table 3), including innate immune responses (158 matched IDs), and overall 441 proteins in hourly samples and 169 proteins in daily samples showing temporal changes post vaccination. Though the potential for longitudinal monitoring of vaccine response using inflammatory saliva markers has been reviewed^62^, MS-based proteomics evaluation of longitudinal saliva responses in pneumonia and PPSV23 vaccination have not been carried out prior to our study, to the best of our knowledge.

In addition to the coupled transcriptome–proteome evaluations (Human Protein Atlas, see above), focused saliva transcriptomics have also been previously evaluated, including through expression microarray analysis^15^, and high throughput sequencing by Spielman et al. who detected the expression of $> 4000$ coding and noncoding genes^17^. In our RNA-seq results we detected 67,319 GENCODE annotation transcripts showing non-zero values for at least 3 timepoints (81,098 observed in at least 1 timepoint), with more than 7493 transcripts detected consistently over 3/4 of the hourly observations, and 8155 over 3/4 of the daily observations.

We have also profiled the RNA of salivary EVs. There has been expansive interest in the diverse RNA content of EVs^63^, including by the Extracellular RNA Communication (ERC) program^64^, for applications in liquid biopsies, as markers in disease states and for cell-free precision medicine diagnostics. EVs are being evaluated as mediators of intercellular communication through molecular transport, offer stable containment of RNA, and can easily be collected for potential diagnostics^63^. EVs have been detected and may move across biofluids, with RNAs from bacteria, fungi, and other species having been reported in human plasma and saliva^65–68^. Ogawa et al. evaluated saliva EV transcriptomes by sequencing^69^, identifying 304 miRNA sequences and 186 non-redundant piRNA sequences across two exosome fractions. Bahn et al. evaluated small RNA in cell free saliva^70^, reporting 127–418 miRNAs, and 32–109 piRNAs with more than 1 RPM detected, and showed overlaps of their miRNA findings in exosomes. Human salivary EVs were also characterized by sequencing by Li et al.^71^, who reported 5649–6813 genes, 482–696 miRNAs, and 190–214 other small RNAs in various library constructions (at at least 1 Read Per Kilobase of transcript, per Million mapped reads, RPKM). Yeri et al. characterized multiple EV profiles in biofluids, including saliva^72^, which are available with multiple samples as part of the exRNA Atlas^68^, processing an average 16 $\times 10^6$ reads and mapping across various biotypes [with $\sim 34\%$ mapping to hg19, 0.02% to piRNA, 1.65% to mature miRNA, including the identification of 336 miRNAs (detected in at least one out of 46 samples, with > 10 counts; 149 miRNAs in at least 50% of the study samples), and a large amount of reads not mapping to human transcriptome]. Godoy et al. compared EV RNA contents across multiple biofluids, including parotid saliva and submandibular and sublingual saliva^67^, and also detected low mapping to human transcriptome (as expected given the microbiome content in saliva), and observed 395+ miRNA ($\ge 10$ reads per million)and < 0.01% piRNA in their saliva samples, while also detecting multiple maps to GENCODE annotations, and other small RNA subtypes. In our mapping, we adopted the same mapping strategy as the exRNA Atlas, implemented by Rozowsky et al.^40^, with similar multi-biotype mapping results, including to non-human exogenous taxa, and our time series included 140–258 miRNAs, 275–589 piRNAs, and 55,499–58,863 GENCODE transcripts that could consistently be evaluated over time (3/4 of the samples in each time frame).

While the content of saliva EVs has been explored, their longitudinal changes, and in particular in response to pneumococcal vaccination (or pneucoccal disease) had not been investigated prior to our study. Additionally, the microbiome EV content is an area of new study, that has not been fully evaluated for its effects in pneumonococcal disease, and there is considerable interest in bacterial EVs (BEVs), for example in the context of cancer^73^. Our goal in this study was to focus on the host, so we did not evaluate the oral microbiome, beyond EV content, though this has been extensively studied, particularly with 16S ribosomal RNA profiling^74,75^, as previously reviewed^76,77^. Recent studies have included longitudinal monitoring of the oral microbiome in the context of oral health: Dzidic et al. investigated the long term effects of colonization during development as associated with tooth decay, associated carries with temporally divergent microbial constitution^78^. Kennedy et al. investigated oral microbiota composition using sequencing in children sampled at 6, 12 and 24 months of age^79^. Kahharov et al. longitudinally profiled the oral microbiome maturation of the Oral Microbiome in caries-free children^80^. Lif Holgerson et al. recently studied the longitudinal development of salivary microbiome in adolescents^81^. In future investigations it will be interesting to study further the interplay between host transcriptome/proteome and oral microbiome in infectious disease, and monitor these in parallel, as we do expect the microbiome to directly affect EV content, and partake in multi-omic interactions potentially modulating immune responses.

In terms of the longitudinal monitoring of individuals over time, profiling multiple omics, such approaches were pioneered with the iPOP study^5^, that measured up to 20 timepoints of multiple blood-based omics in an individual, over a timeframe that included coincidental viral infections, and the onset of type 2 diabetes. David et al.^82^ monitored the daily microbiome in gut and saliva of two individuals, with 274 saliva samples profiled for 16S ribosomal RNA, with their findings indicating that travel and enteric infections affecting community structure. The Pioneer study^7^ which incorporated behavioral coaching to improve clinical biomarkers based on 108 participants’ individual data, including bood-based multiomics profiling, also measured saliva cortisol and dehydroepiandrosterone (dhea) levels for stress assessment every three months. Additional longitudinal studies have focused on host–microbiome characterization of multiple insulin resistant individuals and studying weight gain^8^ and in prediabetics^9^, investigating biological age^10,11^ as well as monitoring of astronauts in the recent NASA twin study^12^. Our study extends these approaches, not only by longitudinally monitoring saliva host transcriptome, proteome and EV content simultaneously, but also in providing dense profiling with hourly sampling. Our study, in conjunction with the previous individual monitoring efforts provide the first steps in personalized wellness monitoring, not only in demonstrating the feasibility of utilizing state-of-the art multiomics technologies, but also providing extensive datasets for modeling temporal processes in direct applications to health, including in our case non-invasive monitoring of immune responses, such as vaccination.

Our study also has limitations: even though we attempted to pair time responses for the hourly data, this is still a single subject case-study (n = 1), and our results will need to be validated. Furthermore, due to limited samples and resources, we did not carry out immune profiling, such as cytokine assays or functional assays to assess the immune acquisition to the different components in the PPSV23 vaccine. We were also unable to obtain blood samples across all the time points, as our focus was a first evaluation of saliva omics. Additionally, our study did not specifically target the salivary microbiome, and the meta-analysis of EV RNA content indicated substantial variability in overall taxa, the composition of which is expected to vary across individuals. In the next stage of our long-term project we have already collected samples from multiple subjects being vaccinated at the same time points with PPSV23 and monitored over multiple timepoints. Given further resources, our goal is to utilize these samples to both validate and extend our findings to also include monitoring of blood components. By comparing the responses in blood and saliva we will be able to assess to what extend saliva may be used as a proxy for blood monitoring, identifying common and different responses in different tissues. Finally, in monitoring total saliva omics in bulk, we are ignoring the multi-cellular composition of saliva. With the availability of single-cell RNA-seq methodology, we anticipate that we will be able to also assess the cell-type-specific response in saliva.

In summary, saliva provides a promising venue for non-invasive diagnostics of immune response. This is particularly important for enhancing our diagnostic capabilities for multiple viral or bacterial responses, particularly in cases where blood may not be easily available, due to technical issues, remote locations (e.g. monitoring active personnel), lack of specialized equipment and healthcare availability (e.g. due to socio-economic factors), patient vulnerability (immuno-compromised, children, and elderly populations). Given the current pandemic (COVID-19), enhancing our diagnostic capabilities has become a high priority. While the utility of saliva for differentiating between different afflictions still needs to be evaluated, our study provides the first steps towards a no-pain no-blood diagnostic process that can greatly enhance our capabilities for universal individualized health care and diagnostics.

## Methods

### Data and protocol availability

Sequencing data reported here are available on Gene Expression Omnibus under accessions GSE108664 (Saliva mRNA-sequencing) and GSE108666 (EV small RNA). Proteomics data are available on MassIVE as part of accession MSV000081869. All scripts and data analysis code utilized in the integrative analysis are available on Zenodo (DOI:10.5281/zenodo.3987587) as online data files (ODFs), in addition to results and methods as referred to in the manuscript.

### Sample collection

Samples were taken in the three time frames hourly for TFH1 and TFH2, and daily for TFD. In TFH2, the PPSV23 pneumococcal vaccine was administered at approximately 10.30 am (between sample collections at 10 and 11 am). Following the vaccination, and after the 24 h monitoring, daily samples were taken for about a month. At each timepoint 5 ml saliva were collected always in the same order: 2 ml in an Oragene (DNAGenotek) tube for RNA sequencing, and 3 ml in a conical tube for EV characterization and mass spectrometry proteomics, as described below. The collected samples were from unstimulated saliva (passive drooling), where the subject was instructed to let saliva collect in the front of their mouth and spit as saliva accumulated over time. The collection took approximately 10 min total per timepoint, for an estimated 500 $\upmu \,\hbox {l/min}$ unstimulated salivary flow rate for the subject. Conical tube samples were immediately stored in a $-20\, ^\circ \hbox {C}$ non-commercial freezer, prior to transfer to the laboratory on ice where they were immediately stored at $-80\, ^\circ \hbox {C}$ on receipt. Oragene tube samples were capped and mixed with the stabilizing liquid that is part of the Oragene tube, and then kept at room temperature until transfer to the lab, where they were processed for RNA-sequencing as described below. Daily samples were all taken at 8 am, to limit variability. Additionally the subject followed the exact same diet and meal timings during TFH1 and TFH2, and had neither meals/drinks nor teeth brushing for at least 30 min prior to TFH1 and TFH2, and 1 h prior to TFD sample donations.

### Saliva sample processing for RNA-sequencing

The saliva samples (2 ml) for RNA processing were collected in Oragene (DNAGenotek) tubes. The samples were incubated at $50\,{^\circ }\hbox {C}$ for 1 h, and stored at $-80^\circ \hbox {C}$. RNA Processing: $500\, \upmu \hbox {l}$ aliquots were incubated at $90\,^{\circ }\hbox {C}$ for 15 min in a heating block and then then cooled to room temperature. $20\,\upmu \hbox {l}$ neutralizer solution were mixed with each aliquot, vortexed and incubated on ice for 10 min, precipitating impurities and inhibitors. The sample was then centrifuged at 13,000 g for 3 min. The supernatant was transferred into a new microcentrifuge tube, 2 volumes of cold 95% EtOH were added and mixed thoroughly, followed by incubation at $-\,20\,^\circ \hbox {C}$ for 30 min. Following centrifugation at 13,000*g* for 3 min, the precipitate was collected. This pellet was dissolved in $350\,\upmu \hbox {l}$ of buffer RLT (RNeasy Micro kit). $350\,\upmu \hbox {l}$ of 70% ethanol were added and mixed. Additional steps followed the RNeasy Cleanup (Qiagen) per manufacturer’s instructions to obtain concentrated RNA.

Libraries were constructed and sequenced by Novogene, using a Eukaryotic directional mRNA library (NEB). cDNA preliminary concentration was quantitated on a Qubit (Life Technologies), an Agilent 2100 Bioanalyzer was used to test the insert size, and Q-PCR was used to quantify the library effective concentration precisely. The cDNA libraries were sequenced on an Illumina HiSeq 4000.

### Saliva EV processing

Saliva samples for EV processing were collected in a conical tube and stored in $-\,80\,^{\circ }\hbox {C}$ on sample receipt. EVs were processed from $500\,\upmu \hbox {l}$ saliva, following centrifugation at 3000*g* for 20 min at $4\,^{\circ }\hbox {C}$. $500\,\upmu \hbox {l}$ of saliva were centrifuged at 3000*g* for 20 min at $4\,^\circ \hbox {C}$ to remove cells and cell debris. ExoQuick-TC Exosome Precipitation Solution (SBI) was added to the supernatant in a 2:1 ratio, and the mixture was refrigerated overnight at $4\,^{\circ }\hbox {C}$. Following incubation samples were centrifuged 1500*g* for 30 min at $4\,^{\circ }\hbox {C}$. The supernatant was aspirated and residual ExoQuick-TC solution was spun down at 1500*g* for 5 min. EV pellets were stored at $-80\,^{\circ }\hbox {C}$. RNA was extracted using the SeraMir RNA Amplification Kit (SBI) per manufacturer’s instructions. The quality of EV RNA was checked using a 2100 Bioanalyzer (Agilent).

Small RNA library preparation was carried out using NEBNext Multiplex Small RNA library prep kit (New England Biolabs) following manufacturer’s instructions. After PCR amplification, quality of libraries was assessed using a high sensitivity DNA kit on a Bioanalyzer (Agilent) according to manufacturer’s instructions. Size selection was performed using 3% agarose dye-free marker H cassettes on a Pippin Prep (Sage Science) following manufacturer’s instructions with a specified collection size range of 125–153 bp. Libraries were further purified and concentrated by ethanol precipitation, resuspended in $10\,\upmu \hbox {l}$ of 10 mM tris-HCl (pH = 8.5) and quantified using a Qubit and a Bioanalyzer. Based on the quantification, equimolar library pools were prepared, quality was assessed as described above and the library was further diluted to 4 nM using 10 mM tris-HCl (pH = 8.5). Pooled libraries were sequenced at a final concentration of 1.2 pM on an Illumina HiSeq 2500 (15-plex, 1 $\times$ 50 bp format).

### Exosome quantitation by ELISA

EV concentrations were quantitated using the EXOCET Exosome Quantitation Assay kit (SBI). EVs from 1 ml of saliva were precipitated using the Exoquick TC protocol (see above). Each exosome pellet was dissolved in $80\,\upmu \hbox {l}$ of lysis buffer and diluted with $80\,\upmu \hbox {l}$ of PBS to be used for duplicate reactions. Samples were then incubated at $37\,^\circ \hbox {C}$ for 5 min to liberate EV proteins, vortexed for 15 s, and centrifuged at 1500*g* for 5 min to remove debris. The supernatant EV protein samples were then assayed on a microtiter plate following the EXOCET kit manufacturer protocol (SBI), including 7 standards and blanks in duplicates. The plate was read using a spectrophotometric plate reader (Bio-RAD) at 405 nm. Spectrophotometry results for standards were used to obtain a linear fit, and sample results were indicative of $\sim 10^9$ EVs/ml (supplementary data on Zenodo).

### EV transmission electron microscopy

Isolated EVs were fixed in 2% paraformaldehyde (PFA) for 5 min. For negative-staining of EVs, $5\,\upmu \hbox {l}$ of the sample solution was placed on a carbon-coated EM grid and EVs were immobilized for 1 min. Next, the grid was transferred to five $100\,\upmu \hbox {l}$ drops of distilled water and letting it for 2 min on each drop. The sample was negative-stained with 1% uranyl acetate. The excess uranyl acetate was removed by contacting the grid edge with filter paper and the grid was air-dried. The grids were imaged with a JEOL 100CXII Transmission Electron Microscope operating at 100 kV. Images were captured on a Gatan Orius Digital Camera.

### Nanoparticle tracking analysis (NTA)

NTA was carried out using the ZetaView (Particle Metrix) following the manufacturer’s instructions. EVs derived from saliva were further diluted 1000- to 5000-fold with PBS for the measurement of particle size and concentration.

### Saliva proteomics (mass spectrometry)

Saliva samples for proteomics processing were collected in a conical tube (same as EVs—see above) and stored in $-80\,^{\circ }\hbox {C}$ on sample receipt. For proteomics processing, the Tandem Mass Tag (TMT) 6-plex kits were used (Thermo). Per sample, $300\,\upmu \hbox {l}$ of saliva were and dissolved in $300\,\upmu \hbox {l}$ lysis buffer (1:1 ratio saliva to lysis buffer to achieve $>\hbox { 2 mg/ml}$ protein concentration). Protein concentration were evaluated using a Qbit (Life sciences). Per timepoint, $100\,\upmu \hbox {g}$ were used, adjusted to a final volume of $100\,\upmu \hbox {l}$ with 100 mM TEAB. The manufacturer’s TMT labeling protocol was then followed to prepare the protein extract (part A, steps 7 onwards, and parts B for protein digestion and C for peptide labeling). Samples were ran through OffGel Fractionation (using an Agilent Offgel 3100 fractionator), and mass spectrometry was carried out with a ThermoFisher Q-Exactive mass spectrometer (www.thermo.com) using a FlexSpray nano-spray ion source. Survey scans were taken in the Orbi trap (70,000 resolution, determined at m/z 200) and the top twelve ions in each survey scan are then subjected to automatic higher energy collision induced dissociation (HCD) with fragment spectra acquired at 35,000 resolution. Additional details are provided in the online experimental protocols on Zenodo (see above).

### Data mapping

#### Total saliva RNA-seq

Fastq files from paired-end sequencing (150 bp paired-end reads) were mapped using Kallisto^32,33^ (with bootstrap sample parameter, -b, set to 100. For annotation, GENCODE^36^ v28 transcripts and genome built GRCh38.p12 were used. The mapping results across timepoints were compiled using sleuth^34^(with DESeq^35^ adjustment of Transcripts per Million). We note that the annotation used gene name concatenated with ‘kind’ information (‘ext_gene’:‘kind’).

The transcriptomics results were imported as OmicsObject constructs in MathIOmica^37^. Zero intensities were tagged as missing values, and intensities with aTPM $< 1$ were set to unity. Time series were constructed only for transcripts for which a signal was detected for at least 3/4 of the time points, and also constant time series were removed.

#### Total saliva proteomics

Proteomics *.raw* mass spectrometry files were analyzed using Proteome Discoverer (Thermo), using UniProt^38^ human proteome database for reference. Mass tolerance was set to 10 ppm for precursor ions, and to 0.02 Dalton for fragment ions. Modifications included cysteine carbamidomethylation (fixed) and N-terminal and lysine TMT 6-plex and methionine oxidation (variable). Furthermore, we allowed for $< 2$ trypsin digestion missed cleavages. Proteins were identified using unique peptides of length $\ge 6$ amino acids. We set FDR $< 1\%$ (strict) and $< 5\%$ (non-strict). For identification, we calculated results for both cases with 1 or 2 unique peptides per protein. We carried out peptide quantitation using unique peptides (reporter ion mass tolerance $< 10\hbox { ppm}$). For protein quantitation, we used medians of peptide ratios.

Multi-consensus reports from each set of technical replicates were constructed and used downstream in MathIOmica to construct an annotated OmicsObject. For each timepoint (sample) a Box-Cox power transformation was first used to transform the data to normal distributions^39^. Time series were constructed only for proteins with at least 2 unique peptides, and for which a signal was detected for at least 3/4 of the time points. Constant time series were also removed.

#### EVs small RNA-seq

Small RNA-seq data from EVs were processed using the Genboree Workbench^41,42,83^ exceRpt pipeline^40^ to assess content by: (1) First removing reads that map to UniVec contaminants, 45S, 5S and mitochondrial rRNAs; (2) mapping reads sequentially to human miRNAs (mirBase), tRNAs (gtRNAdb), piRNAs (piRNABank), GENCODE and circRNAs (cirBase); (3) mapping unmapped reads from (2) to exogenous miRNAs and rRNAs; (4) finally mapping unmapped reads from (3) to all genomes in Ensembl and NCBI. Parameter settings and Genboree output files are available on Zenodo (see data availability).

For each biotype MathIOmica OmicsObject constructs were created. Zero intensities were tagged as missing values, and intensities with aTPM $< 1$ were set to unity. Time series were constructed only for transcripts for which a signal was detected for at least 3/4 of the time points, and also constant time series were removed.

### Temporal analysis and integration

For all mapped data time series were constructed with reference to the first timepoint for TFH1, TFH2 and $\hbox {TF}\Delta$, and with reference to the vaccination day for TFDaily. $\hbox {TF}\Delta$ time series were constructed as paired hourly timepoint intensity differences between TFH2 and TFH1. All series were normalized as vectors to unit length. Time series classification analyses were carried out using MathIOmica as detailed below and in the online Mathematica notebooks^37,84^.

#### Temporal classification details

The time series classification used MathIOmica’s TimeSeriesClassification function with the Method -> “Autocorrelation” setting^37^. Briefly, for a given omic signal *j* with $X_j$ intensities over *N* times we construct a time series $X_j = \left\{ X_j(t_1),X_j(t_2)\ldots ,X_j(t_N)\right\}$. The signal’s periodogram is obtained using a Lomb–Scargle transformation^43–45^ to account for uneven sampling, $P_{LS}$. An inverse Fourier transform on $P_{LS}$ results in the autocorrelations $\rho _j$ for signal *j* as a list for lags 0 to $n=\lfloor N/2\rfloor$, $\rho _j=\left\{ \rho _{\text {j0}},\rho _{\text {j1}},\ldots ,\rho _{\text {jk}},\ldots ,\rho _{\text {jn}}\right\}$. The autocorrelations’ significance (p-value $\le$ 0.01) is assessed by using a list of cutoffs, $\rho _c=\left\{ \rho _{\text {c1}},\rho _{\text {c2},},\ldots ,\rho _{\text {ck}}\right\}$, determined from a null distribution of autocorrelations for each lag. These null distributions are generated from the calculated autocorrelations of simulated random signals that are created by bootstrapping (re-sampling of the original data with replacement). A signal is categorized in a class corresponding to the lowest lag deemed statistically significant, i.e. in class *Lag* *l*, where $l=\text {Min}\left[ \left\{ i:\rho _{\text {ji}}\ge \rho _{\text {ci}}\right\} \right]$, and $i\in {1,\ldots ,k}$. The result is a unique classification for each signal into *Lag* classes, which also ensures that any identified autocorrelation at a particular lag cannot possibly arise due to dependence on autocorrelations at lower lags.

The signals that do not show significant autocorrelation at any lag are checked for sudden signal spikes at any time point, and if so classified as spike maxima or minima. For each signal not showing autocorrelation, $\tilde{X_j}$ the signal maximum, $\max _j=\max \tilde{X_j}$, and minimum, $\min _j=\min \tilde{X_j}$, are calculated across all time points. These values are compared against cutoffs $\left\{ \text {max}_{cn},\text {min}_{cn}\right\}$ generated from bootstrap simulated distributions from the data. If for a signal $\tilde{X_j}$ of length *n*, $\max _j>\text {max}_{cn}$ it is classified in the *SpikeMax* class, or otherwise if $\min _j<\text {min}_{cn}$ it is classified in the *SpikeMin* class. Signals for which the signal intensity does not meet cutoff conditions are not reported.

#### Enrichment analysis

Gene-based over-representation analyses were run using MathIOmica^37^ in Mathematica for GO and Reactome database entries. For miRNA enrichment analysis was run using the miRNA Enrichment Analysis and Annotation tool (miEAA) over-representation tool^85^.

Taxa groups were checked for over-representation using MicrobiomeAnalyst’s^86^’s web interface. Each subgroup and also each aggregate class were tested on multiple levels. The online MicrobiomeAnalyst database used included the following information, to show analyses at three different levels of mixed-level taxons, species-level and strain-level:

- Mixed-Level Taxon sets included the following taxon sets: 1545 associated with host genetic variations, 239 associated with host-intrinsic factors such as diseases, 118 associated with host-extrinsic factors including diet and lifestyle, 446 associated with environmental factors such as drugs, chemical exposures and 53 associated with microbiome-intrinsic factors such as motility, shape, or spore forming.
- Species-level taxon sets included: 61 associated with host-intrinsic factors including diseases, 92 associated with host-extrinsic factors including diet and lifestyle, 7 associated with environmental factors including drugs and chemical exposures.
- Strain-level taxon sets included: 42 associated with host-intrinsic factors including diseases, 50 associated with microbiome-intrinsic factors such as microbe mobility and shape, and 399 associated with environmental factors including drugs and chemical exposures.

The statistically significant ($p < 0.05$) over-representation results are available with the online data on Zenodo (DOI:10.5281/zenodo.3987587).

### Network construction

Weighted expression networks were constructed in which each node represents one molecular species and each edge weight is defined as $w_{ij}= \frac{1}{( d_{ij} +0.0001)}$, where $d_{ij}$ is the Euclidean distance between each pair of nodes $\{i,j\}$, and the offset 0.0001 was added to account for cases where $d_{ij}=0$. Networks were constructed for both the classified TFD data and the TF$\Delta$ data. To account for missing data in the computation of the Euclidean distance, mean imputation was used. Edge selection for the network construction was determined by filtering on one-tailed quantiles *q*(*N*) based on the $w_{ij}$ distribution in a given network *k* with $N_k$ nodes:

1 $$\begin{aligned} q_{k}(N_k) = {\left\{ \begin{array}{ll} 0.8 &{}\quad \text {if } \;N_k < 500\\ 1-\frac{100}{N_k} &{}\quad \text {if }\; N_k\ge 500 \\ \end{array}\right. } \end{aligned}$$

Finally, in the network plots nodes were colored based on the MathIOmica classification group to which they belong.

### Ethical approval

All experimental protocols were approved by the Institutional Review Board under protocol number LEGACY15-071 (15-071) at Michigan State University. All methods were carried out in accordance with the relevant guidelines and regulations. Informed consent was obtained from the participant as per the above protocol.

## Data Availability

Sequencing data reported are available on Gene Expression Omnibus under accessions GSE108664 (Saliva mRNA-sequencing) and GSE108666 (EV small RNA). Proteomics data are available on MassIVE as part of accession MSV000081869. All scripts and data analysis code utilized in the integrative analysis are available on Zenodo (DOI:10.5281/zenodo.3987587) as online data files (ODFs).
